# Reverse Septal Flap Necrosis in Endoscopic Skull Base Reconstruction

**DOI:** 10.1002/alr.70236

**Published:** 2026-08-09

**Authors:** Nana‐Hawwa Abdul‐Rahman, Aileen Cui, Garret Choby, Eric W. Wang, Georgios A. Zenonos, Paul A. Gardner, Carl H. Snyderman

**Affiliations:** ^1^ University of Pittsburgh School of Medicine Pittsburgh Pennsylvania USA; ^2^ Department of Otolaryngology‐Head & Neck Surgery University of Pittsburgh School of Medicine Pittsburgh Pennsylvania USA; ^3^ Department of Neurological Surgery University of Pittsburgh School of Medicine Pittsburgh Pennsylvania USA; ^4^ Department of Neurosurgery New York University Langone Health New York City New York USA

## 1 Introduction

The pedicled nasal septal flap (NSF) revolutionized endoscopic skull base reconstruction by reducing cerebrospinal fluid (CSF) leak rates. Despite its success, NSF harvest leaves a large donor site on the septal cartilage to heal by secondary intention, contributing to postoperative nasal morbidity [1]. The reverse septal flap (RSF) was developed as a secondary flap to cover the exposed septal cartilage, reducing crusting, improving nasal air flow, and minimizing nasal deformities [2]. While the benefits of RSF are well documented, the incidence and risk factors for RSF necrosis have not been systematically studied. The present study aims to evaluate the incidence of RSF necrosis following endoscopic endonasal surgery (EES) and identify associated clinical and operative factors.

**Key Points**

- Reverse septal flap (RSF) necrosis is rare and linked to flap length but not surgical approach.
- Careful reverse septal flap (RSF) design, preserved mucosal perfusion, and avoiding intraoperative trauma are key factors.

## 2 Methods

In this prospective cohort study, 135 patients (with a subset of 29 having complete intraoperative data) underwent EES with NSF and RSF reconstruction between August 2024 and March 2025 at a tertiary academic center. The primary outcome was RSF necrosis, defined by postoperative clinical exam showing epithelial necrosis with tissue loss and exposed perichondrium/cartilage or adherent crust. Covariates include preoperative clinicodemographic variables, intraoperative factors, and postoperative outcomes. Statistical analyses were conducted using STATA, version 18 (StataCorps, College Station, TX, USA), with alpha set at 0.05.

## 3 Results

Of the 135 patients (median age 55 years, 46% females), RSF necrosis occurred in five participants (3.7%) (Table 1). RSF necrosis was managed with partial debridement in clinic and topical application of antibiotic ointment until healing was complete. Necrosis was associated with prolonged healing time (5/5, vs. 6/124, *p* < 0.001). Of the 29 patients with intraoperative data (Table 2), patients who developed RSF necrosis (n = 4) had shorter flap length (median: 2.5 cm [IQR: 2.5–2.5] vs. 3.0 cm [IQR: 2.5‐3], *p* = 0.026). RSF necrosis occurred only in meningioma (2/20) and “other” pathology (3/49), whereas no RSF necrosis was seen in pituitary tumors or chordoma cases (0/66) (*p* = 0.028). Surgical approach (transcribiform vs. all other surgical approaches) was not associated with NSF necrosis. No significant differences were observed in age, sex, BMI, smoking status, major comorbidities, previous sinus surgery or EES, protective nasal sleeve use, RSF vascularity (indocyanine green dye fluorescence), duration of surgery, length of stay, postoperative complications, and ED‐visits or readmission between those with and without RSF necrosis (*p* > 0.05).

**TABLE 1:** Patient demographics and clinical characteristics (*N* = 135).

Variables	All (135)	RSF necrosis, yes (*n* = 5)	RSF Necrosis, No (*n* = 130)	*p* value
	Median (IQR)			
Age (years)	55 (42–69)	60 (57–64)	55 (42–69)	0.42
BMI (kg/m^2^)	31 (25–35)	32 (28–37)	31 (25–34)	0.51
	*N* (%)			
Sex				1.000
Male	73 (54)	2 (40)	60 (46)	
Female	62 (46)	3 (60)	70 (54)	
Pathology type				**0.028**
Meningioma	20 (15)	2 (40)	18 (14)	
Chordoma/pituitary pathology	66 (49)	0 (0)	66 (51)	
Other	49 (36)	3 (60)	46 (35)	
Surgical approach				1.00
Transcribriform	14 (10)	0 (0)	14 (11)	
All other approaches (non‐transcribriform)	121 (90)	5 (100)	116 (89)	
Smoking history				0.662
No	88 (66)	4 (80)	84 (66)	
Yes	45 (34)	1 (20)	44 (34)	
Comorbidities				0.403
No	78 (59)	4 (80)	74 (58)	
Yes	55 (41)	1 (20)	54 (42)	
Previous sinus or EES				0.255
No	106 (80)	3 (60)	103 (81)	
Yes	26 (20)	2 (40)	24 (19)	
Postoperative complications				0.655
No	87 (64)	4 (80)	83 (64)	
Yes	48 (36)	1 (20)	47 (36)	
Healing time				**<0.001**
Normal	124 (92)	0 (0)	124 (95)	
Prolonged	11 (8)	5 (100)	6 (5)	
NSF necrosis				1.000
No	131 (99)	5 (100)	126 (99)	
Yes	1 (1)	0 (0)	1 (1)	
ED visit or readmission				0.537
No	116 (86)	4 (80)	112 (86)	
Yes	19 (14)	1 (20)	18 (14)	

Bold value indicates *p* < 0.05.

**TABLE 2:** Surgical characteristics (*N* = 29).

Variables	RSF Necrosis, yes (*n* = 4)	RSF Necrosis, no (*n* = 25)	*p* value
	Median (IQR)		
Duration of surgery (hours)	7.0 (4.9–7.8)	4.4 (3.8–5.3)	0.408
Length of residual septum	NA	2.5 (2.5–3)	NA
Length of reverse septal flap	2.5 (2.5–2.5)	3 (2.5–3)	**0.026**
Length of stay (days)	3.5 (2–5)	3.5 (2–9.5)	0.605
	*N* (%)		
Nasal sleeve use			0.389
No	0 (0)	4 (16)	
Yes	4 (100)	21 (84)	
Flap vascularity at the end of surgery via ICG			1.000
Grade 0–1	0 (0)	5 (20)	
Grade 2–3	4 (100)	20 (80)	

*Note*: Four out of five patients with RSF necrosis had intraoperative surgical data.

Abbreviation: NA, not reported.

## 4 Discussion

Careful preoperative planning and intraoperative technique have been shown to minimize NSF necrosis, and similar design principles can be applied to optimize RSF survival [3, 4, 5]. The following recommendations are informed by the study's findings and the literature:

1. Maximize RSF length by placing the posterior incision as posterior as possible. This recommendation is supported by our finding that shorter RSF length was an independent predictor of RSF necrosis and is consistent with prior technical descriptions of flap design. Shorter flaps may suffer from increased tension and receive less robust perfusion from the remaining septal mucosa, predisposing the flap to ischemia.
2. Sufficient posterior septal cartilage should be removed to avoid compression of the flap at its flexion point. Enough septum should be resected such that the mucosal flap is slightly longer than the remaining septum [6].
3. Superior and inferior releasing incisions should be no longer than necessary for the transposition of the flap [6].
4. The distal flap should be in apposition to the vestibular mucosa without exposed cartilage at the NSF donor site.
5. Quilting stitches should be avoided since they may create an ischemic zone.
6. Protection of the RSF to avoid injury from passage of instrumentation during surgery is essential [6]. The low incidence of RSF necrosis in our series may be due to universal use of a protective nasal guide of the RSF (SPIWay) during surgery; however, causal conclusions cannot be made because there was no control group.

## 5 Limitations

Only 29 of 135 patients had complete data for the flap‐length subgroup analysis, and key measurements were absent in several cases. Because flap lengths were not documented in the remaining 105 patients, this introduces a high standard error around the flap‐length estimate and raises the possibility of selection bias; thus, these results should be considered hypothesis‐generating rather than definitive. Comparing patients with and without intraoperative documentation on age, pathology type, surgeon/co‐surgeon, prior sinus surgery, surgical approach, and operative duration revealed no significant differences (*p* > 0.05 for all), suggesting the subgroup is broadly representative of the full cohort though the possibility of unmeasured confounding cannot be excluded.

## Funding

No funding was received for this research.

## Ethics Statement

This study was approved by the University of Pittsburgh Institutional Review Board (STUDY20040320).

## Conflicts of Interest

Dr. Snyderman is a consultant for SPIWay, LLC. The remaining authors declare no conflicts of interest.

## Data Availability

The datasets generated during and/or analyzed during the current study are available from the corresponding author upon request.
